# Complete Genome Sequence of a Highly Pathogenic Porcine Reproductive and Respiratory Syndrome Virus Variant Isolated from a Backyard Piglet

**DOI:** 10.1128/genomeA.00123-12

**Published:** 2013-01-31

**Authors:** Kaichuang Shi, Shenglan Mo, Huaxin Huang, Changhua Lin, Chun’ai Tao, Jun Li, Gang Li

**Affiliations:** a Guangxi Center for Animal Disease Control and Prevention, Nanning, China; b Institute of Animal Sciences, Chinese Academy of Agricultural Sciences, Beijing, China

## Abstract

The highly pathogenic porcine reproductive and respiratory syndrome virus (HP-PRRSV) isolates have showed accelerating evolution under the great immune pressure in China in recent years. Here, we report the complete genome sequence of the HP-PRRSV variant GX1001 isolated from a vaccinated backyard piglet.

## GENOME ANNOUNCEMENT

Porcine reproductive and respiratory syndrome virus (PRRSV) is a small, enveloped, single-stranded positive-sense RNA virus belonging to the family *Arteriviridae*. PRRSV has caused immense economic losses to the Chinese swine industry since its first isolation in China in 1996, especially after the emergence of the highly pathogenic PRRSV (HP-PRRSV) variant in 2006, which is characterized by a discontinuous 30-amino-acid deletion (amino acids [aa] 481 and 533 to 561) in its Nsp2-coding region (1). In recent years, under the great immune pressure caused by the extensive use of live-attenuated and inactivated vaccines in China, highly pathogenic porcine reproductive and respiratory syndrome viruses (HP-PRRSVs) have become geared for rapid variation through mutation and recombination, resulting in the emergence of many novel variants (2–6). Here, we report the complete genome sequence of the HP-PRRSV variant GX1001 with unique variations in different regions.

The GX1001 strain was isolated from the lung of an infected backyard piglet, which had been vaccinated with an inactivated vaccine derived from the HP-PRRSV strain JXA1, in a remote village in Guangxi province, southern China, in June 2011. To determine the complete genome sequence of the GX1001 strain, 14 pairs of specific primers, which were designed on the basis of the sequences of PRRSV strains VR-2332 (GenBank accession no. AY150564) and JXA1 (GenBank accession no. EF112445), were used to generate overlapping amplicons by reverse transcription (RT)-PCR, while the 5′ and 3′ termini were obtained by a rapid amplification of cDNA ends using a RACE kit (TaKaRa). The PCR products were purified, cloned into a pMD18-T vector (TaKaRa), sequenced with an ABI3730XL genome sequencer, and assembled with SeqMan software (DNAStar Inc.). DNAStar version 7.0 and Clustal 2.1 were applied to the genomic analysis. As a result, excluding the poly(A) tail, the genome of GX1001 is 15,329 nucleotides (nt) in length, sharing 89.2% and 61.9% nucleotide identity with the American prototype VR-2332 and the European prototype lelystad virus (LV) (GenBank accession no. M96262), respectively. GX1001 has a genome organization similar to that of the representative HP-PRRSV strain JXA1, with 98.9% nucleotide identity and the same discontinuous 30-aa deletion at aa 481 and 533 to 561 in Nsp2, indicating that it belongs to HP-PRRSV. However, GX1001 has 134 nt mutations resulting in 81 aa substitutions, which are distributed in different regions as follows: 76 nt/39 aa in open reading frame 1a (ORF1a), 21 nt/14 aa in ORF1b, 15 nt/14 aa in ORF2, 6 nt/5 aa in ORF3, 10 nt/6 aa in ORF4, 4 nt/3 aa in ORF5, and 2 nt in the 3′ untranslated region (UTR). In addition, GX1001 has unique 6 nt/2 aa insertions, of which 3 nt (CTG) are located at positions 478 to 480 in Nsp9 and 3 nt (TGG) are located at positions 259 to 261 in ORF2a. Therefore, GX1001 acquired variations through mutations and insertions. Since most previous PRRSV strains whose genome sequence data have been deposited in publicly available databases are isolated from intensive pig farms, GX1001 is a rare strain isolated from a backyard piglet and its complete genome sequence will enhance our understanding of the molecular epidemiology and accelerating evolution of HP-PRRSV.

### Nucleotide sequence accession number.

The complete genome sequence of strain GX1001 is available in GenBank under accession number JQ955657.
